# Baicalin promotes apoptosis and inhibits proliferation and migration of hypoxia-induced pulmonary artery smooth muscle cells by up-regulating A2a receptor via the SDF-1/CXCR4 signaling pathway

**DOI:** 10.1186/s12906-018-2364-9

**Published:** 2018-12-12

**Authors:** Xiaoying Huang, Wei Mao, Ting Zhang, Meibin Wang, Xuetao Wang, Yaozhe Li, Lin Zhang, Dan Yao, Xueding Cai, Liangxing Wang

**Affiliations:** Division of Pulmonary Medicine, The First Affiliated Hospital of Wenzhou Medical University, Key Laboratory of Heart and Lung, Wenzhou, Zhejiang, 325000 People’s Republic of China

**Keywords:** Hypoxia, PASMC, Proliferation, Migration, A2a receptor, SDF-1/CXCR4, Baicalin

## Abstract

**Background:**

Baicalin is a flavonoid compound that exerts specific pharmacological effect in attenuating the proliferation, migration, and apoptotic resistance of hypoxia-induced pulmonary artery smooth muscle cells (PASMCs). However, the underlying mechanism has not been fully elucidated yet. Although our previous studies had indicated that activation of A2aR attenuates CXCR expression, little is known about the relationship between A2aR and SDF-1/CXCR4 axis in hypoxic PASMCs. In this study, we aimed to investigate the effect of A2aR on the SDF-1/CXCR4 axis in hypoxic PASMCs, the mechanism underlying this effect, and whether baicalin exerts its protective functions though A2aR.

**Methods:**

Rat PASMCs were cultured under normoxia/hypoxia and divided into nine groups: normoxia, hypoxia, hypoxia + AMD3100 (a CXCR4 antagonist), hypoxia + baicalin, hypoxia + negative virus, normoxia + A2aR knockdown, hypoxia + A2aR knockdown, hypoxia + CGS21680 (an A2aR agonist), and hypoxia + A2aR knockdown + baicalin. Lentiviral transfection methods were used to establish the A2aR knockdown model in PASMCs. Cells were incubated under hypoxic conditions for 24 h. Expression levels of A2aR, SDF-1, and CXCR4 were detected using RT-qPCR and western blot. The proliferation and migration rate were observed via CCK-8 and Transwell methods. Cell cycle distribution and cell apoptosis were measured by flow cytometry (FCM) and the In-Situ Cell Death Detection kit (Fluorescein).

**Results:**

Under hypoxic conditions, levels of A2aR, SDF-1, and CXCR4 were significantly increased compared to those under normoxia. The trend of SDF-1 and CXCR4 being inhibited when A2aR is up-regulated was more obvious in the baicalin intervention group. Baicalin directly enhanced A2aR expression, and A2aR knockdown weakened the function of baicalin. SDF-1 and CXCR4 expression levels were increased in the hypoxia + A2aR knockdown group, as were the proliferation and migration rates of PASMCs, while the apoptotic rate was decreased. Baicalin and CGS21680 showed opposite effects.

**Conclusions:**

Our data indicate that baicalin efficiently attenuates hypoxia-induced PASMC proliferation, migration, and apoptotic resistance, as well as SDF-1 secretion, by up-regulating A2aR and down-regulating the SDF-1/CXCR4 axis.

## Background

Pulmonary arterial hypertension (PAH) is a type of complex cardiopulmonary disease that seriously threatens physical and mental health. It is one of the most common diseases that clinically result in high mortality and disability rates. Among the pathological features, inflammation and pulmonary vascular resistance and remodeling (PVR) are now recognized as the two key factors associated with PAH disease aggravation and death [1]. Abnormal pulmonary arterial smooth muscle cell (PASMC) proliferation, migration, and apoptotic resistance have been emphasized as the cytopathological basis of PVR. Moreover, hypoxia and inflammation are now recognized as the major pathological components responsible for these changes. Many signaling pathways and bioactive factors such as T3K/mTOR signaling [2], Akt [3], and stromal interaction molecule 2 (STIM2) [4] have been implicated in regulating PASMC proliferation and migration, and targeting of these signaling molecules may be a promising strategy for future research.

Stromal cell-derived factor 1 (SDF-1) and the CXCR4 ligand/receptor pair are expressed in a variety of cells and tissues. Originally detected in leukocytes, the SDF-1/CXCR4 axis has been shown to be responsible for the trafficking and homing of hematopoietic progenitors [5]. The SDF-1/CXCR4 axis has been widely reported to have a notable influence on inflammatory reactions, tumor cell invasion [6], heart and vascular diseases [7], nervous system regulation [8], cell growth, and cell cycle modulation [9]. As an important chemokine axis, SDF-1/CXCR4 is pivotal for the recruitment of cells and regulates cell proliferation, migration, cycle, and apoptosis. In addition, many studies have demonstrated the SDF-1/CXCR4 axis to modulate vascular remodeling by mobilization and recruitment of progenitor cells to the pulmonary vasculature, inhibition of SDF-1/CXCR4-attenuated hypoxic PASMC proliferation, and progression of cell cycle. Furthermore, research has suggested that the SDF-1/CXCR4 axis prevents as well as reverses hypoxia-induced cardiopulmonary remodeling by decreasing progenitor cell recruitment to the pulmonary vasculature and decreasing pulmonary vascular cell proliferation [10]. We, therefore, conclude that blocking or down-regulating SDF-1/CXCR4 expression in hypoxic PASMCs might benefit PVR through the attenuation of cell proliferation and migration, and promotion of apoptosis.

Adenosine is an important endogenous signal transduction molecule that plays an important role in the physiology and biochemistry of all organisms. Adenosine, owing to its high affinity for adenosine receptors, has been shown to exert physiological effects, including immune response modulation and vascular reactivity regulation in multiple organs and tissues [11]. Researchers have cloned and characterized four adenosine receptors: adenosine A1, A2a, A2b, and A3. Among these, the A2a receptor (A2aR) has been noted for its widespread expression and complexities of its interaction with other biological factors. Recognized to mediate anti-inflammatory effects, A2aR has been shown to affect proliferation and migration of various types of cells [12]. Previous studies had shown that A2aR contributes to vasodilation by relaxing the vascular smooth muscle cells (VSMCs) and participates in pulmonary arterial pressure and vascular remodeling in vivo in hypoxic A2aR-knockout mice. Zhang et al. [13] found that A2aR activation can suppress the functions of CXCR, thus contributing to the anti-inflammatory activity of adenosine. Therefore, A2aR and SDF-1/CXCR4 axis both participate in vascular remodeling in many tissues and organs [14, 15]. Based on these considerations, we speculated that A2aR might influence PASMC biology and pathology, including proliferation and migration, via regulation of the SDF-1/CXCR4 axis.

Baicalin, a flavonoid compound purified from *Scutellaria baicalensis*, is an emerging multi-therapeutic agent. Several studies have confirmed the anti-inflammatory, anti-immunity, anti-viral, anti-oxidant, and anti-cancer properties of baicalin, besides its ability to affect diastolic blood vessel function and regulate blood pressure [16, 17]. Our previous in-vitro experiments had indicated the association of baicalin with the biological function of A2aR and demonstrated that baicalin intervention effectively attenuates hypoxia-induced PAH in rats by regulating arterial pressure and collagen I and collagen II levels [18]. However, the underlying mechanisms are not yet fully understood. In this study, we hypothesized that baicalin exerts its function by down-regulation of the SDF-1/CXCR4 signaling axis through A2aR stimulation. To validate this hypothesis, rat PASMCs were obtained from SD male rats and cultured under normoxia and hypoxia (to mimic pathological conditions); expression of the A2aR protein was determined right after constructing an A2aR-knockdown model using lentiviral transfection methods. The possible pathways associated with the function of baicalin on hypoxia-induced PASMCs, including A2AR and SDF-1/CXCR4, were detected by quantitative PCR (qPCR) and western blotting. Cell proliferation, migration, apoptosis, and cell cycle were examined to validate the effects of A2aR and baicalin. Our results elucidate the pharmacological action of baicalin, associated with A2aR and the SDF-1/CXCR4 signaling axis, hence implying the potential of baicalin in widespread clinical use.

## Methods

### Experimental animals

PASMCs were obtained from the lung tissues of male Sprague–Dawley (SD) rats (weight: 180 ± 10 g, purchased from Shanghai SLAC Laboratory Animal Co., Ltd., Shanghai, China).

The rats were administered diazepam by SQ injection, with an insulin syringe, for sedation. Thereafter, a correct dose of sodium pentobarbital (200 mg/kg) was intraperitoneally injected in the lower right side of the abdomen. After approximately 15 min, the lung tissues were harvested. Then remove pulmonary artery carefully with ophthalmology scissors. Carefully scraped the artery intima to remove endothelial cells.

All procedures were performed according to the Guide for the Care and Use of Laboratory Animals published by the National Institutes of Health, USA, and approved by the Animal Experimentation Ethics Committee of Wenzhou Medical University.

### Reagents

Baicalin was purchased from Sigma (St. Louis, MO.USA). Adenosine A2A-R lentiviral particles were obtained from Genechem (Shanghai, China). CGS21680 (an A2aR agonist, 2 μmol/L) was obtained from Tocris Cookson (Bristol, UK). AMD3100 (a CXCR4 antagonist, 100 ng/mL) was procured from MCE (Neodesha, KS, USA). Streptomycin, penicillin G, and fetal bovine serum (FBS) were purchased from Gibco BRL Life Technologies (Rockville, MD). Rabbit antibodies against A2aR, SDF-1, CXCR4, GAPDH, and tubulin were obtained from Abcam (Cambridge, UK). Cell counting kit-8 (CCK-8) was procured from Dojindo Laboratories (Kumamoto, Japan). Transwell® chambers were purchased from Corning (Corning, NY, USA). An In-Situ Cell Death Detection (Fluorescein) kit was obtained from Roche (Basel, Switzerland). RNeasy mini kits were obtained from Qiagen (Hilden, Germany).

### Cell culture and treatment

**Fig. 1:**
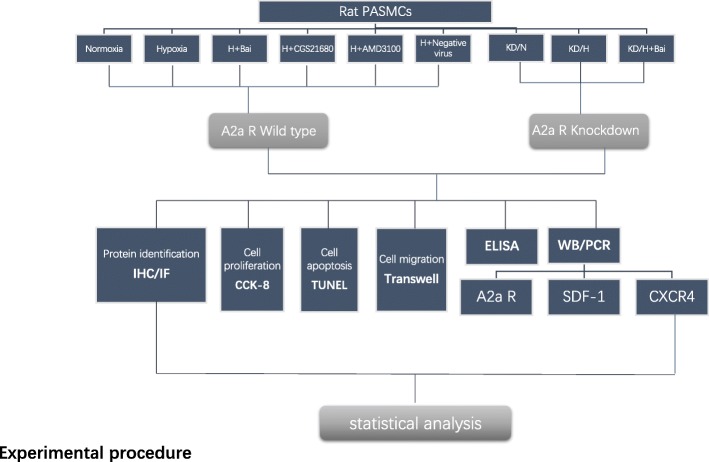
Experimental procedure. Grouping method and Experimental procedure

PASMCs were cultured in Dulbecco’s modified Eagle’s medium (DMEM; Gibco, Grand Island, NY) supplemented with 10% FBS, 100 μg/mL streptomycin, and 100 IU/mL penicillin. Normoxia groups were cultured in a humidified incubator at 37 °C with 21% O_2_, 5% CO_2_, and 74% N_2_. Hypoxia-treated groups were cultured in 5% CO_2_, 5% O_2_, and 90% N_2_ atmosphere of 37 °C. Lentiviral transfection methods were used to create an A2aR knockdown PASMC model. PASMCs from passage 5 were used for this study. Serum-free DMEM was used to starve the cells 24 h before cell synchronization, before each experiment; all experiments were repeated thrice (Fig. 1).

### A2aR knockdown (lentiviral transfection)

The A2aR knockdown model was created by lentiviral transfection, and the virus was used at a multiplicity of infection (MOI) of 15, based on previous experiments [19]. According to the lentiviral transfection protocol, the virus was added to the medium followed by 8-h incubation, and then washed off with DMEM without FBS. The knockdown effect was assessed by RT-qPCR and western blot. The cells were cultured for 12 h and 48 h, as a preliminary experiment to determine the optimal culture period.

### Gene expression analysis

Total RNA was extracted from PASMCs at selected time points using RNeasy mini kits following the manufacturer’s protocol. The extracted RNA was quantified by UV spectrophotometry, and only samples with OD_260_/OD_280_ ratio greater than 1.8 were retained. First-strand cDNA was synthesized using 0.5 μg of RNA. Quantitative PCR was performed using SsoAdvanced™ Universal SYBR® Green Supermix in an CFX384 Touch™ Real-Time PCR Detection System (Bio-Rad, Hercules, CA). GAPDH served as an endogenous control, and all samples were tested in triplicate. The relative fold change was calculated using the 2^–△△CT^ method.

RT-qPCR primers were designed and manufactured by Changfeng Co. (Wenzhou, China). The primer sequences used for RT-qPCR were as follows:

A2aR, forward primer (5′ → 3′): ACTTCTTCGCTTTTGTGTTG, reverse primer (5′ → 3′): GTTGGCTCTCCATCTGCT; CXCR4, forward primer (5′ → 3′): CTACAGCAGCGTTCTCATC, reverse primer (5′ → 3′): TTTTCAGCCAGCAGTTTC; SDF-1, forward primer (5′ → 3′): AAAGAAGCGACAGAAGAAGAG, reverse primer (5′ → 3′): AAGAGGGAGGAGCGAGTT; GAPDH, forward primer (5′ → 3′): AAGAAGGTGGTGAAGCAGG, reverse primer (5′ → 3′): GAAGGTGGAAGAGTGGGAGT.

### Immunohistochemistry (IHC)

IHC was performed to identify PASMC and determine A2aR, SDF-1, and CXCR4 protein expression levels. PASMCs were fixed in 4% paraformaldehyde (Beyotime, Beijing, China) for 15 min at 37 °C and permeabilized using 0.1% Triton X-100 (Beyotime) for 20 min. Thereafter, they were blocked using 5% bovine serum albumin for 1 h at 37 °C and immunostained using the following antibodies for 2 h at 37 °C: anti-α-smooth muscle actin antibody (1:1000; ab5694), anti-adenosine receptor A2a (1:200; ab3461), anti-SDF1 antibody (1:200; ab9797), and anti-CXCR4 antibody (1:500; ab124824). A two-step IHC kit (Zhongshan Jinqiao, Beijing, China) was used, to which 100 μL reaction enhancer was added, and incubated at 37 °C for 20 min. This was followed by incubation with alkaline phosphatase-conjugated goat anti-rabbit antibody (1:1000) for 30 min at 37 °C, counterstaining the nuclei with hematoxylin (1:1000; Beyotime) for 5–8 min, wash with tap water, and incubation again for 20 s for hematoxylin staining (Beyotime). The samples were dehydrated with different concentrations of ethanol and clarified using dimethylbenzene solution. Images were captured with an inverted phase-contrast microscope (SHOIF, shanghai, china).

### Immunofluorescence (IF)

IF assays were conducted to identify PASMCs and determine A2aR, SDF-1, and CXCR4 protein expression levels. The pre-treatment procedure was the same as that used for IHC. IF was performed with anti-α-smooth muscle actin (1:1000, ab5694), anti-adenosine receptor A2a (1:200, ab3461), anti-SDF1 (1:200; ab9797), and anti-CXCR4 (1:500, ab124824) antibodies for 12 h in the dark, followed by incubation with 1:1000 DyLight 488-conjugated goat anti-rabbit IgG (H + L) (Abbkine Inc., North Chicago, IL). The nuclei were stained with 4′,6-diamidino-2-phenylindole (DAPI). Fluorescence images were captured using a fluorescence microscope (Leica DMi8, Wetzlar, Germany). Quantitative analysis was performed using ImageJ analysis software (National Institutes of Health, Bethesda, MD).

### Cell proliferation assay

Cell proliferation was measured using CCK-8 according to the manufacturer’s protocol. PASMCs were plated in 96-well plates at a density of 5 × 10^4^ cells per well. They were first pre-incubated in complete medium at 37 °C with 21% O_2_ and 5% CO_2_. Lentivirus 3–1 was added when the fusion rate was approximately 40%, to construct the A2aR knockdown model. After the knockdown procedure, PASMCs were cultured in FBS-free DMEM for 24 h for synchronization. Thereafter, the cells were administered with different concentrations of SDF-1 (50, 100, 150, or 200 ng/mL), AMD3100 (100 ng/mL), CGS21680 (2 μmol/L), and baicalin (40 μmol/L). The cells were continuously cultured for 24 h, treated with 10 μL/well CCK-8 solution for 1.5 h, and results were checked every 30 min. Cell proliferation curves were plotted using the absorbance data at 450 nm.

### Cell apoptosis assay

Cells were seeded onto glass slides in 6-well microplates at a concentration of 5 × 10^4^ cells/well. After pretreatment, cell slides were fixed in 4% phosphate-buffered paraformaldehyde for 10–15 min. Proteinase K (Thermo Fisher Scientific, Waltham, MA) was used to remove all endogenous nucleases before using the fluorescein TUNEL kit, according to the manufacturer’s instructions. A positive control group was established with 100 μL DNase 1 (Thermo Fisher Scientific), and a negative control group was established by adding only 50 μL of fluorescein-labeled dUTP solution to the slides. The slides were incubated for 1 h at 37 °C temperature, away from direct sunlight. DAPI was used to stain the cell nuclei (blue). After the slides were mounted using Antifade Mounting Medium, PASMCs were observed and counted under a fluorescence microscope. The excitation wavelength range used was 450–500 nm, and the emission wavelength range was 515–565 nm (green fluorescence).

### Cell migration assay

For the Transwell migration assays, 5 × 10^4^ PASMCs were suspended in cell migration medium (DMEM + 10% BSA + 1% penicillin/streptomycin). Cells were plated in triplicate on the upper well chamber. In this study, we used modified Boyden chambers with 8-μm pores in 24-well plates. The wells were loaded with complete medium with a concentration gradient of SDF-1 (50, 100, 150, and 200 ng/mL) or baicalin (40 μmol/L) as a chemoattractant stimulus.

Samples were cultured under normoxic or hypoxic conditions. After 48 h of incubation, PASMCs on the upper chamber surface of the Boyden chamber side were gently wiped off using a cotton swab. PASMCs that migrated to the bottom surface were fixed in 70% ethanol for 10 min before treatment with crystal violet solution (Beyotime) for visualization. Six random fields were selected for every sample and cells were counted under a microscope.

### Cytokine assay

Secretion of SDF-1 was determined using enzyme-linked immunosorbent assay (ELISA) kits according to the manufacturer’s protocols. Briefly, SDF-1 protein was extracted from the cell supernatant by centrifuging at 1000×g for 20 min. Optical density of each well was recorded at 450 nm.

### Cell cycle distribution

Cell cycle stages were detected by flow cytometry (FCM) following propidium iodide (PI) staining. PASMCs were seeded at a concentration of 5 × 10^4^ cells/well and incubated under normoxia or hypoxia for 24 h. The culture medium was washed off with phosphate-buffered saline (PBS), and PASMCs were collected after trypsinization by centrifugation for 5 min at 1000 rpm. Pre-cooled PBS was used for centrifugation to separate the flocculated cell debris. Cells were then fixed in pre-cooled methanol (− 20 °C) at 4 °C for 30 min and centrifuged in pre-cooled PBS again, after which 1% RNase (Thermo Fisher Scientific) in 200 μL PBS was added to each tube. Samples were incubated for 30 min at 37 °C in a moist atmosphere before PI staining. FCM assays (BD Biosciences, San Diego, CA) were performed to assess the cell cycle, and data were analyzed using FlowJo 7.6 software (BD Biosciences).

### Western blot analysis

Total proteins were extracted from PASMCs using One-Step Animal Tissue/Cell Active Protein Extraction buffer. Cells were seeded in 6-well plates, and after pre-treatment, each sample was separated by 10% sodium dodecyl sulfate polyacrylamide gel electrophoresis (SDS-PAGE) using a Bio-Rad Electrophoresis System. After gel electrophoresis, the proteins were transferred to a nitrocellulose membrane (SDF-1 using a micro-molecule membrane). Membranes were blocked with 5% skim milk for 1 h, washed with Tris-buffered saline with Tween 20 (TBST), and incubated with a 1:1000 dilution of rabbit primary antibodies, including A2aR (1:1000, ab3461), SDF-1 (1:1000, ab9797), CXCR4 (1:1000, ab124824), and GAPDH, at 4 °C overnight. They were then incubated with a 1:10000 dilution of goat anti-rabbit secondary antibody (Cell Signaling Technology, Danvers, MA) at room temperature for 1 h. A Bio-Rad ChemiDoc MP was used to examine the membranes, and analysis was performed using Image Lab software (Bio-Rad).

### Statistical analysis

All experiments were repeated in triplicate, and the data are presented as the mean ± standard deviation (SD). Data were analyzed with one-way analysis of variance (ANOVA) followed by the least significant difference (LSD) test. All statistical analyses were performed using SPSS version 21.0 and GraphPad Prism 7. Differences were considered statistically significant at *P* < 0.05.

## Results

### Positive expression of A2aR protein in PASMCs under normoxia and hypoxia

**Fig. 2:**
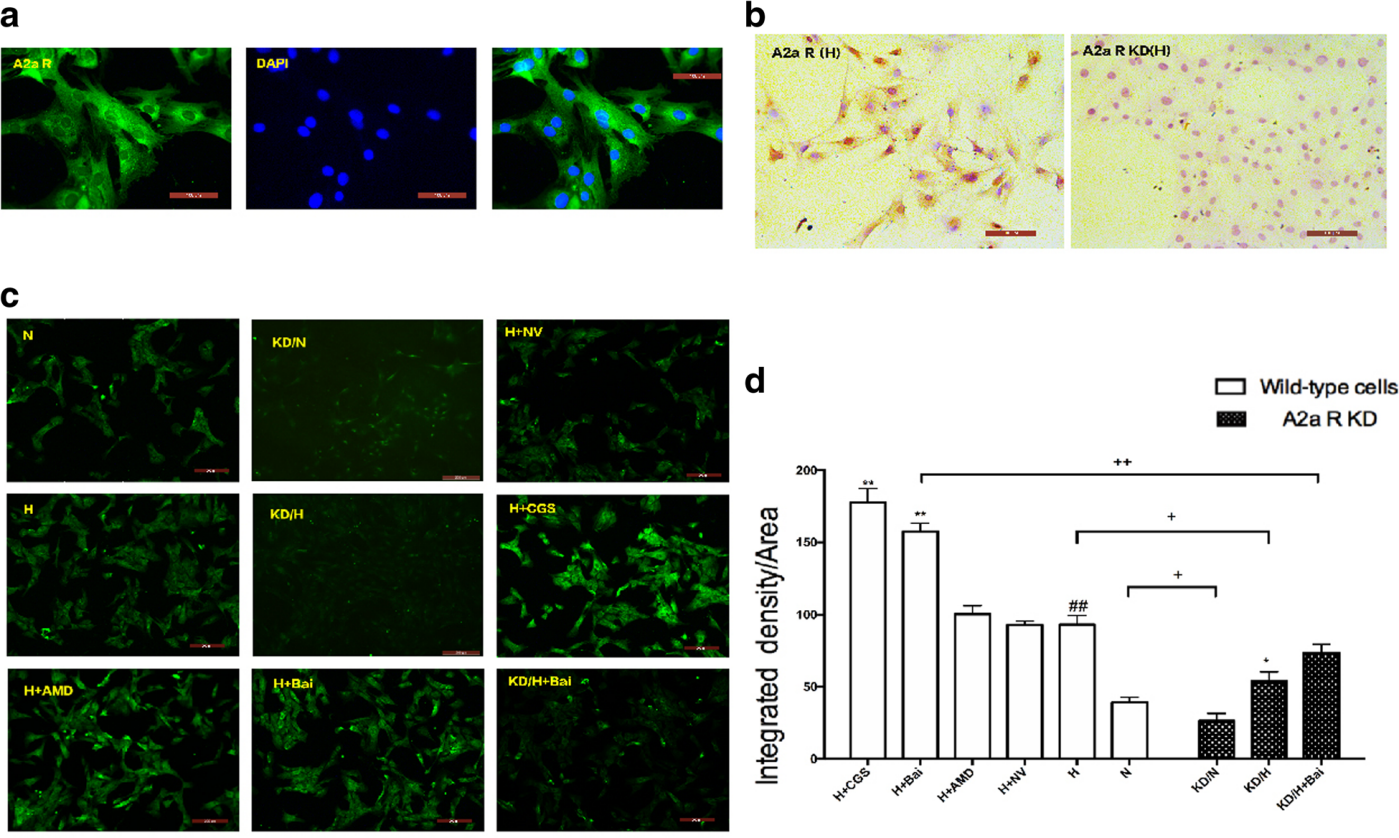
Expression of A2aR protein was upregulated in hypoxic PASMCs. **a** Fluorescence-staining to identify A2aR expression. **b** Immunohistochemical results of hypoxia group and hypoxia A2aR knockdown group. **c** Semi-quantitative analysis of A2a R expressions by immunofluorescence stain. **d** Comparison of integrated density among all groups. Data are presented as mean ± SD. # *P* < 0.05, ##*p* < 0.01 vs normoxia group, * *P* < 0.05 **P < 0.01 vs hypoxia group; + *p* < 0.05, ++ *p* < 0.01 Comparison between groups. *n* = 3

Results of IHC and IF showed that A2aR protein is mainly expressed in the cell membrane and cytoplasm, as evidenced by yellow staining, in PASMCs (Fig. 2a and b). Semi-quantitative detection by IF staining and fluorescence microscopy indicated that hypoxia significantly enhanced the expression of A2aR (*P* < 0.05) compared to normoxia (Fig. 2c and d).

### Promotion of SDF-1 secretion and SDF-1/CXCR4 expression in hypoxic PASMCs

**Fig. 3:**
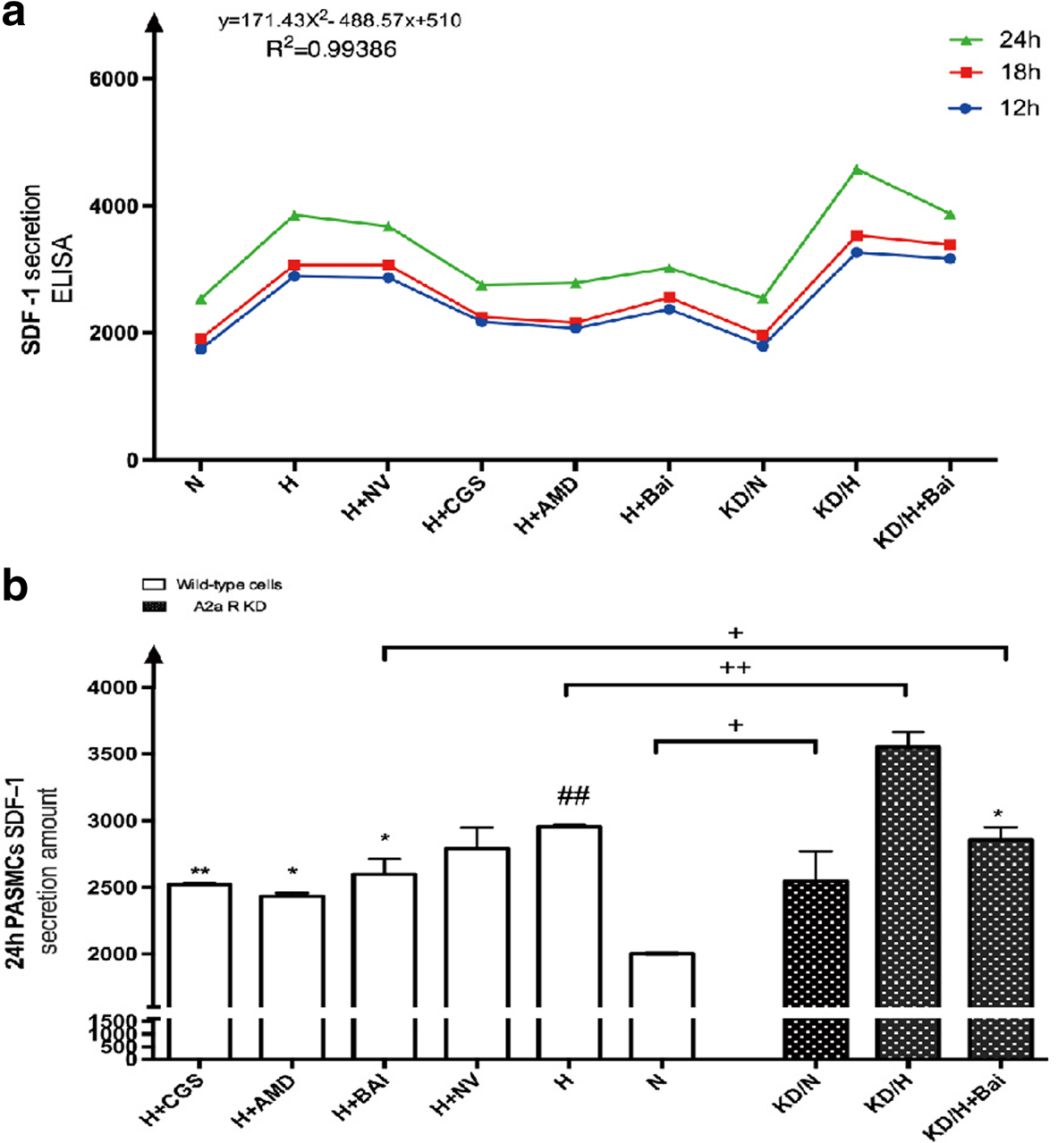
A2aR stimulation and Baicalin attenuated hypoxia induced PASMCs SDF-1 secretion detected by ELISA method. **a**: SDF-1 secretion amount increased with hypoxia treatment time prolonged. **b**: Comparison among groups at 24H time point. Data are presented as mean ± SD. # *P* < 0.05, ##*p* < 0.01 vs normoxia group, * *P* < 0.05 **P < 0.01 vs hypoxia group; + *p* < 0.05, ++ *p* < 0.01 Comparison between groups. *n* = 6

We measured SDF-1 levels in cellular supernatant to investigate the effect of hypoxia on SDF-1 secretion. ELISA results showed that SDF-1 secretion was up-regulated with prolonged hypoxia intervention time (*P* < 0.05). This confirmed that hypoxia enhanced the chemokine factor SDF-1 expression, which may trigger pathological changes in cells. All groups were subsequently compared at the 24-h time point, and results showed that, compared to that in the hypoxia group, A2aR knockdown increased SDF-1 secretion (*P* < 0.01), whereas A2aR up-regulation significantly decreased SDF-1 secretion (*P* < 0.01). Collectively, these findings indicated that hypoxia plays a key role in SDF-1 secretion, whereas A2aR stimulation could reverse this effect (Fig. 3).

### Enhancement of A2aR expression by baicalin and exertion of its function partially through A2aR

**Fig. 4:**
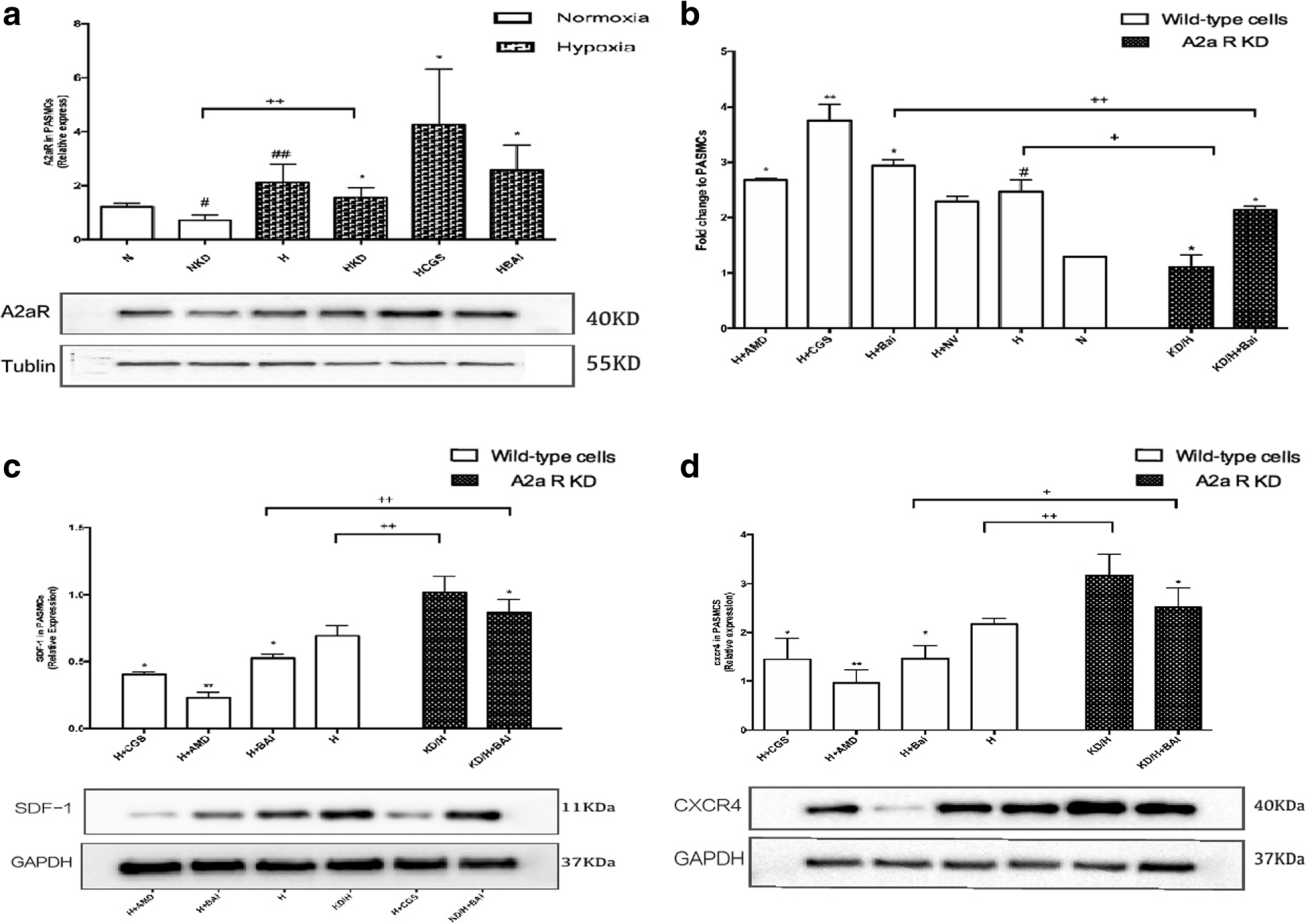
Baicalin intervention enhanced A2aR expression. **a**: Images and quantitave analysis of A2aR expression in PASMCs by western blot assay. Tubulin served as an internal control. **b**: Rt-qPCR indicated that baicalin upregulated A2aR mRNA in hypoxic PASMCs. **c**: SDF-1 expression increased in KD/H + Bai group compared with H + Bai group. **d**: CXCR4 expression increased in KD/H + Bai group compared with H + Bai group. # *P* < 0.05, ##*p* < 0.01 vs normoxia group, * *P* < 0.05 **P < 0.01 vs hypoxia group; + *p* < 0.05, ++ *p* < 0.01 Comparison between groups. *n* = 3

We investigated the effect of baicalin on A2aR expression in PASMCs using IF staining, RT-qPCR, and western blotting. Results suggested that baicalin effectively increases A2aR expression in PASMCs exposed to hypoxia (*P* < 0.05) (Fig. 4a and b). Moreover, we established an A2aR knockdown model in PASMCs to investigate the relationship between baicalin and A2aR. Baicalin was administered in A2aR-knockdown PASMCs under hypoxia and compared to baicalin treatment alone. Results showed that SDF-1 and CXCR4 protein expression levels increased (*P* < 0.05) after A2aR knockdown, thereby indicating that absence of A2aR weakened the protective effects of baicalin (Fig. 4c and d).

### Correlation between up-regulation of A2aR and inhibition of the SDF-1/CXCR4 pathway

**Fig. 5:**
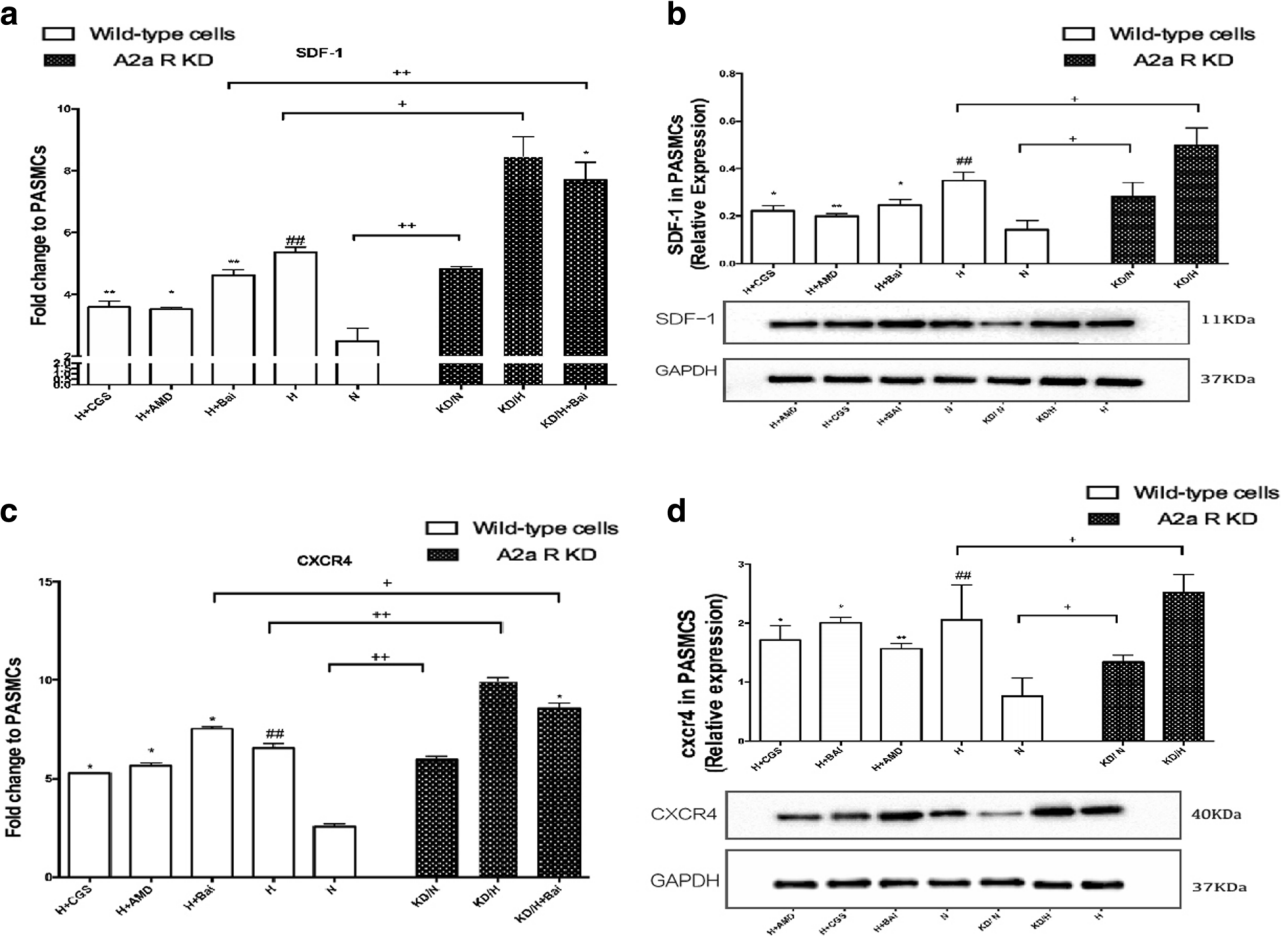
The upregulation of A2aR inhibited hypoxia induced SDF-1/CXCR4 pathway. **a**: Rt-qPCR detected fold change of SDF-1 in each group. **b**: Images and quantitave analysis of SDF-1 expression in PASMCs by western blot assay. GAPDH served as an internal control. **c**: Rt-qPCR detected fold change of SDF-1 in each group. **d**: Images and quantitave analysis of CXCR4 expression in PASMCs by western blot assay. GAPDH served as an internal control # *P* < 0.05, ##*p* < 0.01 vs normoxia group, * *P* < 0.05 **P < 0.01 vs hypoxia group; + *p* < 0.05, ++ *p* < 0.01 Comparison between groups. *n* = 3

To confirm whether A2aR up-regulation inhibited the hypoxia-induced SDF-1/CXCR4 pathway, mRNA and protein expression levels of these molecules were examined (Fig. 5). While the mRNA levels of SDF1 and CXCR4 were reduced in the CGS21680(2 μmol/L) and baicalin groups (*P* < 0.05), protein expression of SDF-1 and CXCR4 was increased in the A2aR knockdown group, compared to the levels in the wild-type groups (*P* < 0.05). These data indicated attenuation of the SDF-1/CXCR4 pathway by the up-regulation of A2aR.

### A key role of hypoxia-induced SDF-1/CXCR4 pathway in regulating cell proliferation, migration, apoptosis, and cell cycle distribution

The CXCR antagonist AMD3100 and a concentration gradient of SDF-1 (50, 100, 150, and 200 ng/mL) was used to investigate the effect of SDF-1/CXCR4 axis on cell proliferation, migration, apoptosis, and cell cycle distribution.

**Fig. 6:**
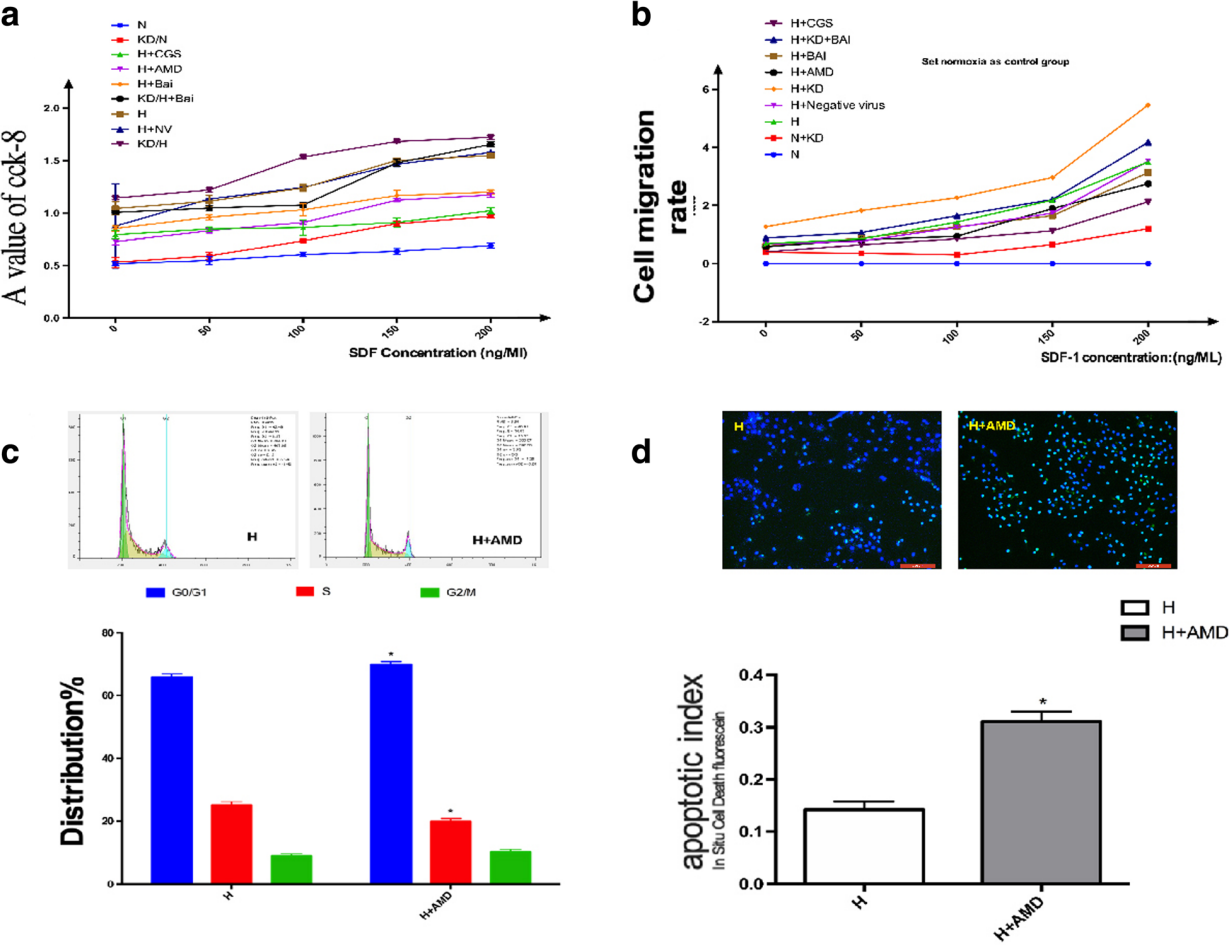
Proliferation and migration ability of PASMCs under different concentration of SDF-1 detected by CCK-8 and Transwell assay. **a**: Proliferation abilities increased with the increase of SDF-1 concentration. **b**: Migration abilities increased with the increase of SDF-1 concentration. **c**: Blockage of SDF-1/CXCR4 axis alters cell cycle distribution. **d**: Blockage of SDF-1/CXCR4 axis attenuates apoptosis-resistance in hypoxic PASMCs. # *P* < 0.05, ##*p* < 0.01 vs normoxia group, * *P* < 0.05 **P < 0.01 vs hypoxia group; + *p* < 0.05, ++ *p* < 0.01 Comparison between groups. *n* = 3

As shown in (Fig. 6a and b), increased concentration of SDF-1 remarkably promoted in-vitro proliferation and migration abilities of PASMCs (*P* < 0.05), compared to the hypoxia group, exhibiting a linear correlation. In contrast, AMD3100 treatment decreased the in-vitro proliferation and migration abilities (*P* < 0.05). Results of In-Situ Cell Death Detection Kit and FCM analysis (shown in Fig. 6c and d) revealed that pre-incubation with AMD3100 attenuated apoptotic resistance in hypoxic PASMCs. These data suggested a key role played by the SDF-1/CXCR4 pathway in regulating cell proliferation, migration, apoptosis, and cell cycle distribution.

### Comparison of the proliferation ability of PASMCs among all groups

**Fig. 7:**
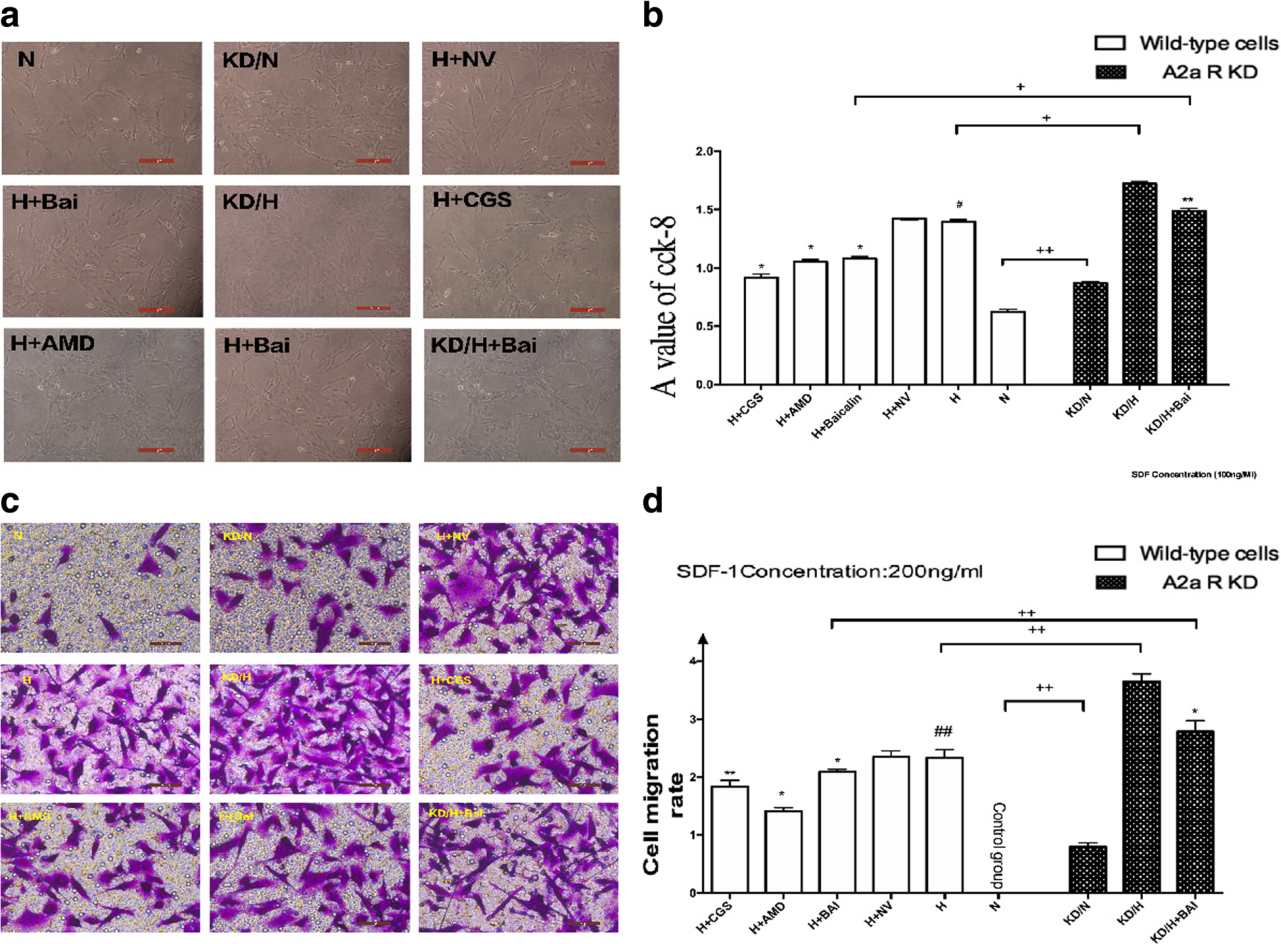
A2aR upregulation and Baicalin attenuated hypoxia induced PASMCs proliferation and migration. **a**: Cell density in each group as viewed under a microscope. **b**: CCK-8 values in each group. **c**: Migrated PASMCs in each group as viewd under a microscope. **d**: Cell migration rate in each group detected by Transwell assay. # *P* < 0.05, ##*p* < 0.01 vs normoxia group, * *P* < 0.05 **P < 0.01 vs hypoxia group; + *p* < 0.05, ++ *p* < 0.01 Comparison between groups. *n* = 3

As shown in Fig. 7a and b, compared to the normoxia group, hypoxic PASMCs exhibited a stronger proliferation ability (*P* < 0.05); the A2aR knockdown group also exhibited an increase in cell proliferation ability (*P* < 0.05). In contrast, the CGS21680 and baicalin groups exhibited decreased cell proliferation ability compared to the hypoxia control group (*P* < 0.05). Finally, relative to the hypoxia + baicalin group, hypoxia + A2aR knockdown + baicalin treatment resulted in reduced proliferation ability, indicating that up-regulation of A2aR suppresses hypoxia-induced cell proliferation. Thus, the pharmacological action of baicalin involves regulation of the in-vitro proliferation ability of PASMCs, probably via A2aR.

### Comparison of the migration ability of PASMCs among all groups

Transwell assays showed that the migration ability of hypoxic PASMCs was enhanced compared to that in the normoxia group (*P* < 0.05). Moreover, knockdown of A2aR reduced migration ability relative to that in the wild-type group (*P* < 0.05). In addition, the migration ability was significantly reduced in the CGS21680 and baicalin groups (*P* < 0.05). Compared to the hypoxia + baicalin group, migration ability of the hypoxia + A2aR knockdown + baicalin group was clearly reduced (*P* < 0.05). The data showed that baicalin attenuates cell migration by up-regulating A2aR (Fig. 7c and d).

### Comparison of cell cycle and apoptotic rate of PASMCs among all groups

**Fig. 8:**
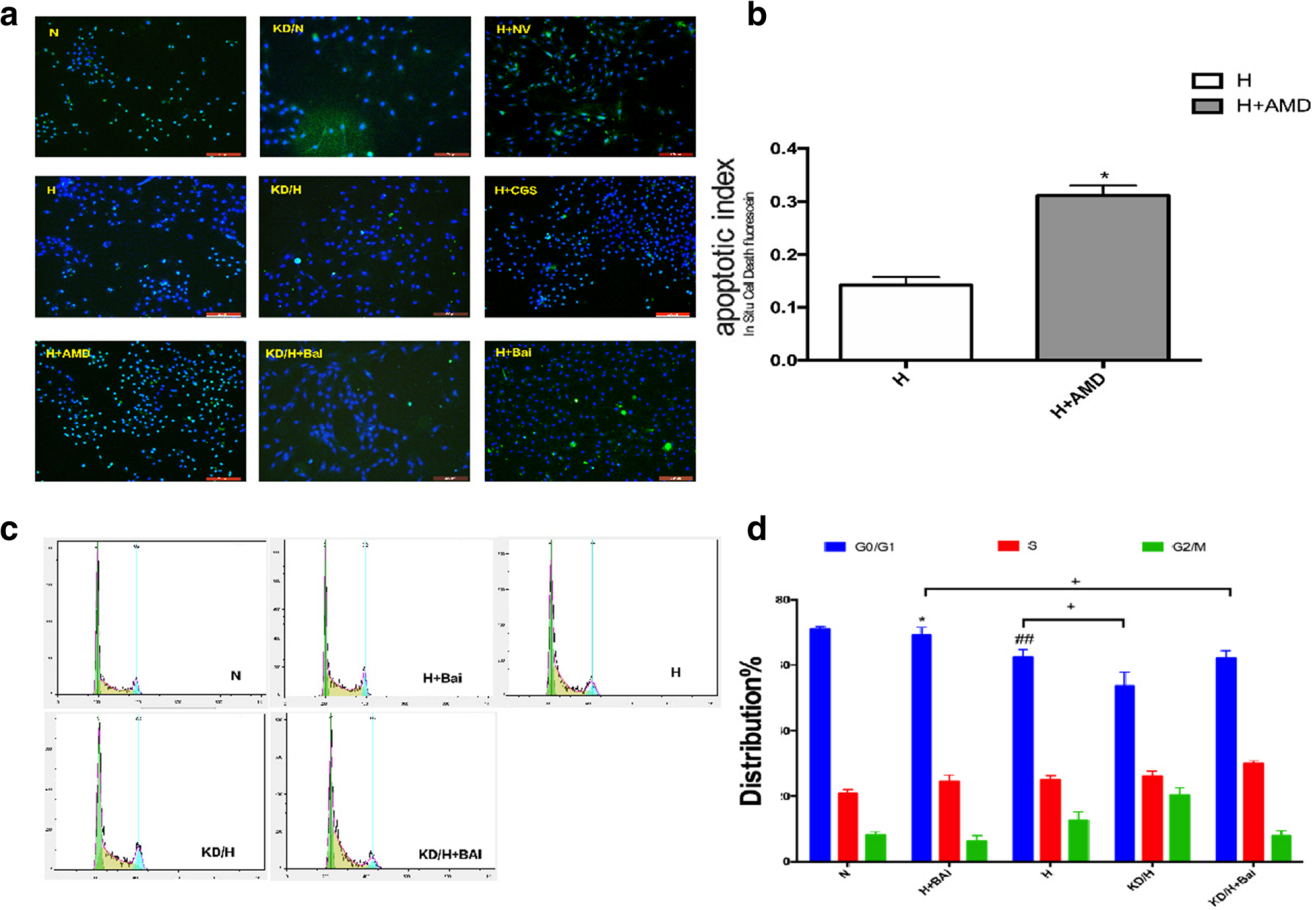
A2aR and Baicalin enhanced cell apoptosis and altered cell cycle. **a**: Fluorescent staining can detect the apoptotic cells. **b**: Comparison of apoptosis rates among all groups. **c**: Cell cycle observed by flow cytometry. **d**: Proportion of different cell cycles in each group. # *P* < 0.05, ##*p* < 0.01 vs normoxia group, * *P* < 0.05 **P < 0.01 vs hypoxia group; + *p* < 0.05, ++ *p* < 0.01 Comparison between groups. *n* = 3

To further explore the regulatory mechanism of A2aR and baicalin, we examined apoptosis using fluorescent staining. As shown in Fig. 8a and b, presence of apoptotic cells was exhibited by dense, green, granular fluorescent staining. Therefore, A2aR knockdown significantly inhibited apoptosis in PASMCs (*P* < 0.05), whereas CGS21680 or baicalin treatment clearly promoted PASMC apoptosis (*P* < 0.05). Compared to hypoxia + baicalin group, hypoxia + A2aR knockdown + baicalin group exhibited a reduced apoptotic rate (*P* < 0.05).

We next investigated the effect of A2aR and baicalin on cell cycle in PASMCs using FCM (Fig. 8c and d). Compared with those of normoxia group, PASMCs of hypoxia group showed a prolonged S-phase, and the G0/G1 ratio was significantly reduced. This effect was more obvious in the A2aR knockdown group, but was attenuated in the CGS21680 group, since the S-phase was shorter and G0/G1 ratio was increased. The above results suggested that A2aR up-regulation by baicalin can promote apoptosis and inhibit proliferation in hypoxic PASMCs, and that baicalin exerts it functions, at least partially, via A2aR.

## Discussion

PAH is characterized by PVR and pulmonary neomuscularization. PVR involves changes in the intima, media, and adventitia, often with the interplay of inflammatory cells [20]. The main cytopathological basis of PVR is PASMC proliferation, migration, and apoptotic resistance, causing the thickening of blood vessel walls and arterial stenosis. This leads to the development of chronic pulmonary artery pressure elevation. A number of studies have confirmed that chronic, sustained, and intermittent hypoxia can induce a series of structural and functional changes in PASMCs, including abnormal differentiation, cell proliferation, migration, and apoptotic resistance [21, 22].

The chemokine SDF-1 and its receptor CXCR4 ligand have been proven to be closely related to cell proliferation, adhesion, organ-specific transfer, and immune response in various cells [23]. Recent studies have reported that the SDF-1/CXCR4 axis plays a regulatory role in pulmonary fibrosis, acute lung injury, lung cancer, and other diseases. Furthermore, researchers have found that antagonism of the SDF-1/CXCR4 axis attenuates right ventricular remodeling [24], thus providing clues regarding the targeting of SDF-1/CXCR4 axis in hypoxic PASMCs.

Some reports have shown that hypoxia, as an initial factor, changes the cell growth microenvironment and directly promotes cell proliferation [25]. In our study, western blot analysis revealed that hypoxic treatment induced high expression of SDF-1 and CXCR4 proteins in PASMCs. Studies have confirmed that high expression of hypoxia inducible factor-1 (HIF-1) under hypoxia increases the expression of CXCR4, forming a hypoxia response element to co-mediate multiple reactions under hypoxia [26]. In this study, we measured SDF-1 concentration in the cell supernatants, results of which confirming that hypoxia promotes SDF-1 secretion in PASMCs. Longer durations of continuous hypoxic treatment increased the amount of SDF-1 secreted. Moreover, compared to normoxia, hypoxia promoted the proliferation of PASMCs. Incubation with the CXCR4-specific antagonist AMD3100 effectively inhibited the hypoxia-induced proliferation of PASMCs, confirming that hypoxia can promote PASMC proliferation via activation of the SDF-1/CXCR4 chemokine axis. In addition, cell migration has been shown to be a key factor in PVR. The SDF-1/CXCR4 axis has been demonstrated to promote cell migration and invasion in various organs and under a variety of pathological conditions [27, 28]. Our study confirmed that a hypoxic environment increases PASMC migration rate and that the chemokine SDF-1 effectively promotes cell migration in a dose-dependent manner. Pre-incubation with AMD3100 significantly inhibited the activity of SDF-1/CXCR4 axis. Together, these results suggested that the SDF-1/CXCR4 axis plays a role in up-regulating proliferation and migration in hypoxic PASMCs.

Apoptosis is another important cytological factor in the pathology of vascular remodeling. Hypoxia has been shown to lead to apoptotic resistance, resulting in the increased proliferation of smooth muscle cells and further pathological deterioration. The process of cell apoptosis is quite complex and involves a variety of pathways. Liu et al. [29] revealed the close relation of SDF-1/CXCR4 axis to cell apoptosis. In this study, cell apoptosis was confirmed by fluorescent cell staining. Suppression of apoptosis in PASMCs was found to be attenuated by incubation with the A2aR agonist CGS21680, SDF-1/CXCR4 inhibitor AMD3100, and baicalin. In summary, the in-vitro data confirm that hypoxia promotes the expression of SDF-1/CXCR4 chemokine axis in PASMCs, thereby leading to PASMC proliferation and migration. Thus, blockade or attenuation of the SDF-1/CXCR4 axis may be a key target for preventing PVR.

Adenosine is an important immunomodulatory factor, and, combined with the adenosine receptors (A1, A2, etc.), it can play a role in regulating metabolism and diastolic blood vessels [30]. In pulmonary circulation, adenosine mainly combines with the A2 receptor on the surface of smooth muscle cells, thereby activating adenosine cyclase, which promotes the formation and release of cAMP and induces the relaxation of smooth muscle. Some studies have found that the pathological conditions of COPD, along with the continual increase in pulmonary vascular tension, may lead to an increase in the expression of some core biological regulatory factors. Ramkumar et al. [31] confirmed that under hypoxic conditions, HIF-1 and kappa-B modulate the expression of adenosine receptors. Based on these findings, in this study, we established an A2aR knockdown cell line using lentiviral transfection and constructed a hypoxia cell model, through continuous 5% O_2_ culture, to explore the effect of A2aR on the cell biology of hypoxic PASMC proliferation, migration, and secretion.

Results of this study were consistent with those of previous reports, indicating that stimulation of A2aR in hypoxic PASMCs attenuated the hypoxic up-regulation of SDF-1/CXCR4. Based on the results from CCK-8 and Transwell assay, A2aR was found to inhibit hypoxia-induced PASMC proliferation and migration and promote PASMC apoptosis by directly down-regulating the SDF-1/CXCR4 axis. Our findings suggested that the activation of A2aR could be used as a potential therapeutic target to attenuate PVR.

As a flavonoid extracted from the plant *Scutellaria baicalensis* Georgi, baicalin has recently gained wide attention for its anti-tumor, vasodilating, anti-inflammatory, anti-viral, and anti-diabetes effects [32, 33]. A number of studies have confirmed that baicalin can promote apoptosis and reduce cell proliferation, migration, and invasion in various cells [34]. Huang et al. [35] confirmed that baicalin exerts these effects by inhibiting TGF-β1 signaling. Li et al. [36] found that baicalin can exert its anti-inflammatory effects by inhibiting CXC chemokines (including SDF-1/CXCR4 and IL-8). However, validation of the relationship between baicalin and A2aR would require further studies. In this study, western blot assays showed that baicalin enhanced A2aR expression in hypoxic PASMCs, whereas its administration in the A2aR knockdown group revealed that the absence of A2aR weakens the pharmacological functions of baicalin. These data indicate that baicalin exerts its protective functions, at least partially, through A2aR. Therefore, we can conclude that baicalin efficiently regulates cell proliferation, migration, and apoptosis and may contribute to reversing the development and complications of PVR via A2aR stimulation.

## Conclusions

In this study, SDF-1 secretion and SDF-1/CXCR4 expression were increased in hypoxic PASMCs, thereby enhancing PASMC proliferation, migration, and apoptotic resistance, as well as changing the cell cycle distribution. These effects can be attenuated by the up-regulation of A2aR. Baicalin intervention partially down-regulated SDF-1/CXCR4 by up-regulating A2aR. We, therefore, conclude that baicalin may reverse hypoxia-induced patterns of proliferation, migration, apoptotic resistance, and change in the cell cycle distribution in PASMCs.

**Fig. 9:**
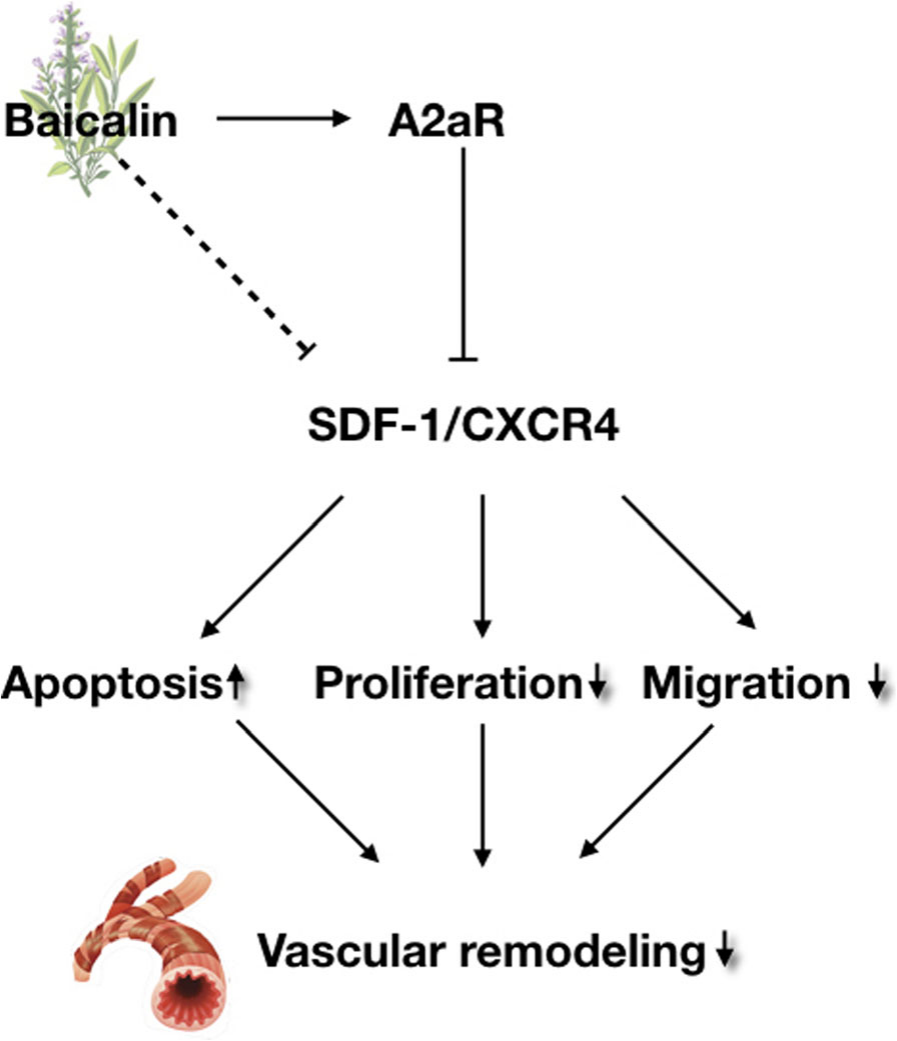
The signaling pathways of this experiment. Baicalin exerted protective effects against hypoxic PASMCs via the upregulation of A2aR expression and downregulation of SDF-1/CXCR4 axis

In conclusion, our study suggests that baicalin promotes hypoxic PASMC apoptosis and inhibits proliferation, migration, and SDF-1 secretion via the down-regulation of SDF-1/CXCR4 signaling pathway through adenosine A2aR stimulation. Our findings many help in providing experimental evidence for application of baicalin in PVR treatment (Fig. 9).

## Abbreviations

A2aR: A2A adenosine receptor
BSA: Bovine serum albumin
CCK: Cell Counting Kit
DAPI: 4′,6-diamidino-2-phenylindole
DMEM: Dulbecco’s modified Eagle’s medium
ELISA: Enzyme-linked immunosorbent assay
FBS: Fetal bovine serum
FCM: Flow cytometry
HIF-1: Hypoxia inducible factor-1
IHC: Immunohistochemistry
PASMC: Pulmonary artery smooth muscle cell
PBS: Phosphate-buffered saline
PHA: Pulmonary arterial hypertension
PI: Propidium iodide
PVR: Pulmonary vascular remodeling
SDF-1: Stromal cell-derived factor 1
TUNEL: Terminal deoxynucleotidyl transferase dUTP nick-end labeling

## References

[1] Tuder RM (2017). Pulmonary vascular remodeling in pulmonary hypertension. Cell Tissue Res.

[2] Zhang H, Gong Y, Wang Z, Jiang L, Chen R, Fan X (2014). Apelin inhibits the proliferation and migration of rat PASMCs via the activation of PI3K/Akt/mTOR signal and the inhibition of autophagy under hypoxia. J Cell Mol Med.

[3] Yi B, Cui J, Ning JN, Wang GS, Qian GS, Lu KZ (2012). Over-expression of PKGIα inhibits hypoxia-induced proliferation, Akt activation, and phenotype modulation of human PASMCs: the role of phenotype modulation of PASMCs in pulmonary vascular remodeling. Gene.

[4] Song S, Carr SG, Mcdermott KM, Rodriguez M, Babicheva A, Balistrieri A (2018). STIM2 (stromal interaction molecule 2)-mediated increase in resting cytosolic free Ca2+ concentration stimulates PASMC proliferation in pulmonary arterial hypertension. Hypertension.

[5] Petit I, Jin D, Rafii S (2007). The SDF-1-CXCR4 signaling pathway: a molecular hub modulating neo-angiogenesis. Trends Immunol.

[6] Teicher BA, Fricker SP (2010). CXCL12 (SDF-1)/CXCR4 pathway in cancer. Clin Cancer Res.

[7] Cheng M, Huang K, Zhou J, Yan D, Tang YL, Zhao TC (2015). A critical role of Src family kinase in SDF-1/CXCR4-mediated bone-marrow progenitor cell recruitment to the ischemic heart. J Mol Cell Cardiol.

[8] Jiang Z, Zhou W, Guan S, Wang J, Liang Y (2013). Contribution of SDF-1α/CXCR4 signaling to brain development and glioma progression. Neurosignals.

[9] Huang M, Li Y, Zhang H, Nan F (2010). Breast cancer stromal fibroblasts promote the generation of CD44+CD24- cells through SDF-1/CXCR4 interaction. J Exp Clin Cancer Res.

[10] Young KC, Torres E, Hatzistergos KE, Hehre D, Suguihara C, Hare JM (2009). Inhibition of the SDF-1/CXCR4 axis attenuates neonatal hypoxia-induced pulmonary hypertension. Circ Res.

[11] Jacobson KA, Gao ZG (2006). Adenosine receptors as therapeutic targets. Nat Rev Drug Discov.

[12] Nowak M, Lynch L, Yue S, Ohta A, Sitkovsky M, Balk SP (2010). The A2aR adenosine receptor controls cytokine production in iNKT cells. Eur J Immunol.

[13] Zhang N, Yang D, Dong H, Chen Q, Dimitrova DI, Rogers TJ (2006). Adenosine A2a receptors induce heterologous desensitization of chemokine receptors. Blood.

[14] Xu MH, Gong YS, Su MS, Dai ZY, Dai SS, Bao SZ (2010). Absence of the adenosine A2A receptor confers pulmonary arterial hypertension and increased pulmonary vascular remodeling in mice. J Vasc Res.

[15] Yu L, Hales CA (2011). Effect of chemokine receptor CXCR4 on hypoxia-induced pulmonary hypertension and vascular remodeling in rats. Respir Res.

[16] Chan FL, Choi HL, Chen ZY, Chan PS, Huang Y (2000). Induction of apoptosis in prostate cancer cell lines by a flavonoid, baicalin. Cancer Lett.

[17] Krakauer T, Bao QL, Young HA (2001). The flavonoid baicalin inhibits superantigen-induced inflammatory cytokines and chemokines. FEBS Lett.

[18] Liu P, Yan S, Chen M, Chen A, Yao D, Xu X (2015). Effects of baicalin on collagen Ι and collagen ΙΙΙ expression in pulmonary arteries of rats with hypoxic pulmonary hypertension. Int J Mol Med.

[19] Gui D, Cui Z, Zhang L, Yu C, Yao D, Xu M (2017). Salidroside attenuates hypoxia-induced pulmonary arterial smooth muscle cell proliferation and apoptosis resistance by upregulating autophagy through the AMPK-mTOR-ULK1 pathway. Bmc Pulm Med.

[20] Shimoda LA, Laurie SS (2013). Vascular Remodeling in Pulmonary Hypertension. J mol med.

[21] Guan Z, Shen L, Liang H, Yu H, Hei B, Meng X (2017). Resveratrol inhibits hypoxia-induced proliferation and migration of pulmonary artery vascular smooth muscle cells by inhibiting the phosphoinositide 3-kinase/protein kinase B signaling pathway. Mol Med Rep.

[22] Zeng Y, Liu H, Kang K, Wang Z, Gang H, Zhang X (2015). Hypoxia inducible factor-1 mediates expression of miR-322: potential role in proliferation and migration of pulmonary arterial smooth muscle cells. Sci Rep.

[23] Qian Y, Wang X, Lv Z, Guo C, Yang Y, Zhang J (2016). MicroRNA-126 is downregulated in thyroid cancer cells, and regulates proliferation, migration and invasion by targeting CXCR4. Mol Med Rep.

[24] Dai S, Yuan F, Mu J, Li C, Chen N, Guo S (2010). Chronic AMD3100 antagonism of SDF-1alpha-CXCR4 exacerbates cardiac dysfunction and remodeling after myocardial infarction. J Mol Cell Cardiol.

[25] Deng B, Du J, Hu R, Wang AP, Wu WH, Hu CP (2016). MicroRNA-103/107 is involved in hypoxia-induced proliferation of pulmonary arterial smooth muscle cells by targeting HIF-1β. Life Sci.

[26] Guan G, Zhang Y, Lu Y, Liu L, Shi D, Wen Y (2015). The HIF-1Î±/CXCR4 pathway supports hypoxia-induced metastasis of human osteosarcoma cells. Cancer Lett.

[27] Jun LI, Guo W, Xiong M, Han H, Chen J, Mao D (2015). Effect of SDF-1/CXCR4 axis on the migration of transplanted bone mesenchymal stem cells mobilized by erythropoietin toward lesion sites following spinal cord injury. Int J Mol Med.

[28] Li M, Sun X, Liang M, Lu J, Zhang W, Xiao M (2017). SDF-1/CXCR4 axis induces human dental pulp stem cell migration through FAK/PI3K/Akt and GSK3β/β-catenin pathways. Sci Rep.

[29] Liu X, Duan B, Cheng Z, Jia X, Mao L, Fu H (2011). SDF-1/CXCR4 axis modulates bone marrow mesenchymal stem cell apoptosis, migration and cytokine secretion. Protein & Cell.

[30] He W, Mazumder A, Wilder T, Cronstein BN (2013). Adenosine regulates bone metabolism via A1, A2A, and A2B receptors in bone marrow cells from normal humans and patients with multiple myeloma. FASEB J.

[31] Ramkumar V, Hallam DM, Nie Z (2001). Adenosine, oxidative stress and cytoprotection. Jpn J Pharmacol.

[32] Ikemoto S, Sugimura K, Yoshida N, Yasumoto R, Wada S, Yamamoto K (2000). Antitumor effects of Scutellariae radix and its components baicalein, baicalin, and wogonin on bladder cancer cell lines. Urology.

[33] Wang H, Zhang Y, Bai R, Wang M, Du S (2016). Baicalin attenuates alcoholic liver injury through modulation of hepatic oxidative stress, inflammation and sonic hedgehog pathway in rats. Cell Physiol Biochem.

[34] Wang XF, Zhou QM, Du J, Zhang H, Lu YY, Su SB (2013). Baicalin suppresses migration, invasion and metastasis of breast cancer via p38MAPK signaling pathway. Anti Cancer Agents Med Chem.

[35] Huang S, Chen P, Shui X, He Y, Wang H, Zheng J (2014). Baicalin attenuates transforming growth factor-β1-induced human pulmonary artery smooth muscle cell proliferation and phenotypic switch by inhibiting hypoxia inducible factor-1α and aryl hydrocarbon receptor expression. J Pharm Pharmacol.

[36] Li BQ, Fu T, Dongyan Y, Mikovits JA, Ruscetti FW, Wang JM (2000). Flavonoid Baicalin inhibits HIV-1 infection at the level of viral entry. Biochem Biophys Res Commun.

