# Correction: Baicalin promotes apoptosis and inhibits proliferation and migration of hypoxia-induced pulmonary artery smooth muscle cells by up-regulating A2a receptor via the SDF-1/CXCR4 signaling pathway

**DOI:** 10.1186/s12906-023-03958-1

**Published:** 2023-04-18

**Authors:** Xiaoying Huang, Wei Mao, Ting Zhang, Meibin Wang, Xuetao Wang, Yaozhe Li, Lin Zhang, Dan Yao, Xueding Cai, Liangxing Wang

**Affiliations:** Division of Pulmonary Medicine, The First Affiliated Hospital of Wenzhou Medical University, Key Laboratory of Heart and Lung, Wenzhou, Zhejiang, 325000 People’s Republic of China

**Correction: BMC Complement Med Ther 18, 330 (2018)**

10.1186/s12906-018-2364-9

Following publication of the original article [1], the authors identified an error in Fig. 7. The correct figure is given below.

**Fig. 7:**
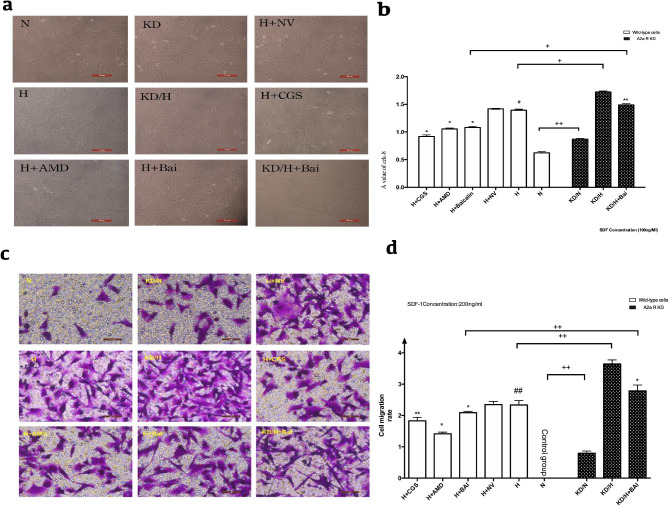
A2aR upregulation and Baicalin attenuated hypoxia induced PASMCs proliferation and migration. **a**: Cell density in each group as viewed under a microscope. **b**: CCK-8 values in each group. **c**: Migrated PASMCs in each group as viewd under a microscope. **d**: Cell migration rate in each group detected by Transwell assay. # *P* < 0.05, ##*p* < 0.01 vs. normoxia group, * *P* < 0.05 **P < 0.01 vs. hypoxia group; + *p* < 0.05, ++ *p* < 0.01. Comparison between groups. n = 3.

The original article [1] has been corrected.
